# Novel Antigenic Variant Infectious Bursal Disease Virus Outbreaks in Japan from 2014 to 2023 and Characterization of an Isolate from Chicken

**DOI:** 10.3390/pathogens13121141

**Published:** 2024-12-23

**Authors:** Mari Takahashi, Shiori Oguro, Atsushi Kato, Soma Ito, Nobuyuki Tsutsumi

**Affiliations:** 1 Nisseiken Co., Ltd., 9-2221-1 Shin-machi, Ome 198-0024, Tokyo, Japan; 2 Nippon Institute for Biological Science, 9-2221-1 Shin-machi, Ome 198-0024, Tokyo, Japan

**Keywords:** genogroup, infectious bursal disease virus, isolation, novel antigenic variant, pathogenicity, phylogenetic tree analysis

## Abstract

Novel antigenic variant strains of the infectious bursal disease virus (IBDV) classified into genogroup A2d have been found in the western part of Japan since 2017. Novel antigenic variant IBDVs now occur in higher frequencies in poultry houses and have been detected in the eastern part of Japan, indicating the spread of IBDVs despite the usual IBDV vaccination. We isolated a novel antigenic variant IBDV, designated as the B2977CE2C3 strain. The B2977CE2C3 strain had two genogroup A2d specific amino acids—lysine and isoleucine, at 221 and 252 aa—along with the other genogroup A2 common amino acids in the projection domains of the VP2 protein corresponding to the virus-neutralizing epitopes and viral pathogenicity. Experimental infection of the B2977CE2C3 strain did not produce any apparent clinical signs in the specific-pathogen-free chickens during the observation period (21 days), but atrophy of the bursa of Fabricius (BF) was apparent. The mean BF to the body weight ratio was 0.35 in negative control chickens at 21 days post-infection (pi) but 0.06 in the B2977CE2C3 infected group. An extremely high copy number of the IBDV genome (>10^8^ copies/µL) was observed in the BF at 3 days pi, while a high copy number of the IBDV genome (>10^6^ copies/µL) was observed in the thymus, spleen cecal tonsil, and bone marrow even though macroscopic lesions were not apparent in these organs.

## 1. Introduction

Infectious bursal disease (IBD) is a highly contagious disease that causes severe immunosuppression in young chickens and has brought considerable economic losses to the poultry industry worldwide because it increases susceptibility to other infectious diseases and the failure of effective vaccination [1]. The causative agent of IBD is the infectious bursal disease virus (IBDV). Chickens are primarily infected with IBDV orally or nasally [2]. IBDV transmits via the fecal-oral/nasal route and spreads through the poultry house.

IBDVs are classified into two serotypes (I and II), of which only serotype I IBDV is pathogenic to chickens. Serotype II IBDV is isolated in turkeys. IBDV belongs to the genus *Avibirnavirus* in the family *Birnaviridae*. It is characterized by two segmented double-strand RNAs, A (3.2 kb) and B (2.8 kb), in a non-enveloped icosahedral capsid virion [3]. Segment A has two partially overlapping open reading frames (ORFs), one of which encodes viral proteins VP2 (48 kDa), VP3 (32 kDa), and VP4 (24 kDa) in a polyprotein manner, while the other ORF encodes the VP5 (17 kDa) protein. Segment B encodes the VP1 (90 kDa) protein, the viral RNA-dependent RNA polymerase/replicase. The VP2 protein is a major structural component of the IBDV virion and is a major host-protective antigen that induces the neutralizing antibodies against IBDV [4]. A hypervariable region (HVR) was found in nucleotide positions 616 and 1090 of the *VP2* gene corresponding to the amino acid positions 206 and 350 of the VP2 protein. This region is known to form the four loop structures projected outside of the VP2 protein, namely, Projection (P)_BC_ (204–236 aa), P_DE_ (240–265 aa), P_FG_ (270–293 aa), and P_HI_ (305–337 aa) [5,6]. As this region is closely related to viral antigenicity and pathogenesis, it is considered a primary determinant of viral virulence and antigenic variation [7]. It has been suggested that the hyper amino acid changes are caused by immune selection pressure for escape variants in chickens immunized against IBDV. Strains of serotype I IBDV have been classified into eight genogroups based on the sequence diversity of the HVR in segment A, termed genogroups A1 to A8 [8,9]. The VP3 protein binds to the double-strand RNA genome to form the inner capsids of the virus and acts like a scaffold protein during IBDV assembly [10]. The VP4 protein acts as a viral protease to cleave the VP2-VP4-VP3 polyprotein. The VP5 protein is a non-structural protein, and its function is not well understood, but it is thought to act like the leucine-rich repeat family protein, such as the host Toll-like receptor [11].

The main target of infection is IgM-positive B-lymphocytes (lymphoblasts). Clinically significant IBD occurs in chickens between three and six weeks of age when the bursa of Fabricius (BF) is mostly filled with the lymphoblastoid population [12]. IBDV kills the lymphoblasts in the BF, destroys the lymphoid follicles of the BF, and depletes the circulating B-cells in the secondary lymphoid tissues. Subclinical IBD occurs in IBDV-infected chicks before three weeks of age when the lymphoblast population is not high in the BF, and consequently, the BF is insufficient for generating clinical signs, leading to systemic effects [13]. Even during a subclinical infection, however, IBDV causes severe damage to lymphoblasts in the BF. Chickens over eight weeks of age are usually resistant to IBDV infection, but infection by very virulent IBDV (vvIBDV) strains can occur in exceptional cases [14,15].

Wild-type IBDVs are known to replicate not only in lymphoblasts but also in macrophages [16] and monocytes [17] of chickens. Several host cell receptors for IBDV have been suggested, namely chicken Hsp90 [18], a4b1 integrin [19], surface IgM l light chain [20,21], CD74 isoform li-2 [22], and CD44 [23]. Of these, overexpression of chicken CD44 on the nonpermissive human 293T cell line did not support IBDV replication but enabled entry of the virus into the cells. This indicated the need for host factors, which have a role in the post-entry uncoating process as the molecular chaperones. A combination of host cell receptors on the surface and the molecular chaperones in the cytoplasm is thought to determine the IBDV cell tropism [23].

The IBDV was first isolated in the United States in the late 1950s and was subsequently reported worldwide. The first strains are now called classical IBDV strains. Vaccines against classical IBDV were then developed and widely used in chicken farms. In the mid-1980s, an antigenic variant IBDV strain that could escape the classical IBDV-based vaccination and a vvIBDV strain that caused over 60% mortality emerged in North America and Europe [15]. Since 2017, the emergence of novel antigenic variant IBDV strains that escaped the immunity provided by the vvIBDV-based vaccines but were characterized by not causing mortality while still causing severe atrophy of the BF were reported in China [24,25,26]. The novel antigenic variant IBDV has become prevalent in China. The occurrence of novel antigenic variant IBDVs was subsequently reported in South Korea [27], Japan [28], and Malaysia [29]. Isolation of novel antigenic variant IBDV strains has been reported beyond eastern and southern Asian countries in Egypt [30] and Argentina [31].

According to the genogroup classification based on the sequence diversity of the HVR in segment A, classical IBDV, antigenic variant IBDV, and vvIBDV strains are classified into A1, A2, and A3 genogroups, respectively. A1 and A2 genogroups are further subclassed into A1a to A1b [8] and A2a to A2d [32], respectively. The novel antigenic variant IBDV strains are classified into A2d [33]. The A4 genogroup contains the distinct IBDV strains found in South America. The A5 genogroup contains IBDV strains found in Mexico, which are thought to have emerged by the genetic recombination between classical IBDV and antigenic variant IBDV. The A6 and A7 genogroups incorporate IBDV strains found in Italy and Australia, respectively. The A8 genogroup is a newly proposed IBDV classification and contains A7 variant IBDV strains found in Australia [8].

In this study, we report that the IBD cases caused by the novel antigenic variant IBDV occurred in the western part of Japan in 2017. We obtained the IBDV (B2977 isolate) from one of the affected bursal homogenates of chickens in 2020 and amplified it in the chorioallantoic cavity of specific-pathogen-free (SPF) chicken eggs. The IBDV B2977CE2C3 strain was finally obtained by propagating in chickens. We found the novel antigenic variant IBDV specific amino acids in the VP2 protein by sequencing VP2-VP4-VP3 polyprotein and VP5 ORF genes on the segment A RNA. Significant BF atrophy by a novel antigenic variant, the B2977CE2C3 strain, was confirmed by experimental SPF chicken infection.

## 2. Materials and Methods

### 2.1. Detection of IBDV Strains in Japan

Dead broiler chickens of 35- to 45-day-old were sent from the broiler houses, where the presence of IBD was suspected from the given circumstantial evidence and used to clarify the recent status of IBD in Japan. The BFs taken from chickens were used to see if IBDV was positive or not by amplifying the DNA fragment containing the HVR using RT-PCR (see Section 2.4 below). In total, 122 RT-PCR-positive samples obtained between 2014 and 2023 were studied (Table 1).

### 2.2. Chickens and Eggs

Experimental animal research using SPF chickens was carried out according to the regulations and guidelines established by the Nippon Institute of Biological Science (NIBS) Animal Care and Use Committee (Approval no 02E-2021, 019B-2021, 003B-2022, and 011B-2022). All animal procedures were performed according to the principles outlined in the Japanese Animal Welfare and Management Act. SPF white leghorn chickens (Line-M) and embryonated hen eggs laid by SPF chickens were obtained from the Kobuchizawa facility of Nisseiken Co., Ltd. (Hokuto, Japan).

### 2.3. Viral Nucleic Acid Extraction

Tissue samples, such as cecal tonsil, thymus, spleen, BF, and bone marrow, were temporarily stored at −80 °C for subsequent IBDV RNA isolation. The frozen tissue samples were thawed immediately before homogenization. Samples were weighed, and nine volumes (convert 1 g to 1 mL) of the phosphate-buffered saline containing penicillin and streptomycin (1000 μg/mL each) were added. Samples were homogenized in a tissue blender and followed by centrifugation (9800× *g* for 10 min). Supernatants were designated as 10% (*w*/*v*) tissue homogenates. Viral RNA (60 μL) was extracted from 140 μL of the 10% (*w*/*v*) tissue homogenates using the QIAamp Viral RNA Mini kit (Qiagen K.K., Tokyo, Japan) according to the procedure indicated by the manufacturer.

### 2.4. IBDV Detection Using PCR and Segment A Sequencing of IBDV

An RT-PCR was used to amplify the 474 bp-cDNA fragment corresponding to nucleotide positions from 607 to 1080 on the *VP2* gene using the PrimeScript One Step RT-PCR Kit Ver.2 (Takara Bio Inc., Kusatsu, Japan) with the forward primer (P2.3; 5′-CCCAGAGTCTACACCATA-3′) and the reverse primer (RP5.3; 5′-TCCTGTTGCCACTCTTTC-3′) according to the previous study [34]. The thermal conditions were as follows: a single cycle of the reverse transcription for 30 min at 50 °C, inactivation of the reverse transcriptase for 2 min at 94 °C and polymerase amplification of cDNA by 35 amplification cycles of 15 s at 94 °C, 30 s at 52 °C and 45 s at 72 °C, and a final extension for 5 min at 72 °C. The lengths of amplified cDNA fragments were confirmed using 2.0% (*w*/*v*) agarose gel electrophoresis in Tris–Acetate–EDTA (TAE) buffer. If necessary, an amplified fragment was purified using the PCR cleaning kit (NucleoSpin Gel and PCR Clean-up Kit, Macherey-Nagel-Takara Bio Inc., Kusatsu, Japan) and was sequenced by ordering the sequencing service provider (Fasmac Co., Ltd., Atsugi, Japan). The IBDV genogroup was then classified using the hypervariable region of the *VP2* gene (VP2-HVR, amino acids 210–350) within the 474 bp-cDNA fragment. To identify the copy number of the IBDV genome, a quantitative PCR (qPCR) was performed by modifying the method described by Tomás et al. [35]. The primer pairs (forward; 5′-ARACAAYGGGCTGACGGC-3′and reverse; 5′-GTTAWCTCGYTGGTYGGGAA-3′) and the probe (5′-FAM-AGRTTGAATG-GCATRAGR-BHQ1-3′) were used to detect both the genogroup A2 B2977CE2C3 strain sequence and the genogroup A1 IBDV strain sequence. Moreover, the TaqMan Fast Virus 1-Step Master Mix reagent (Thermo Fisher Scientific Inc., Tokyo, Japan) was used for amplification by the StepOnePlus real-time thermal cycler (Thermo Fisher Scientific Inc.). A 72 bp-cDNA fragment corresponding to the nucleotide positions from 964 to 1035 on the *VP2* gene was amplified by qPCR. A 474 bp-cDNA fragment amplified as described above was purified using a PCR cleaning kit. Then it was quantitated using OD260 of the spectrophotometer DS-11 (DeNovix Inc., Wilmington, DE, USA) and used as the copy number standards (10^1^, 10^2^, 10^3^, 10^4^, 10^5^, 10^6^, 10^7^, and 10^9^ copies/μL). The segment A sequence of the novel variant IBDV was determined by primer walking and was registered in the DNA database (GenBank accession number: LC844776).

### 2.5. Calculation of EID_50_ in Embryonating Hen Eggs

Titrations of IBDV were made by inoculating 10-fold serial dilutions of viral samples through the chorioallantoic cavity route. Briefly, five SPF 10-day-old embryonating eggs were inoculated with 0.1 mL of a viral sample per dilution. All embryos were incubated for seven days at 37.0 °C. Eggs were candled daily, and the chick embryo was taken out from the egg and checked macroscopically at the end of the incubation period. Titers were expressed as the 50% egg embryo infective dose (EID_50_)/μL as the reciprocal of the highest virus dilution at which 50% of eggs are probabilistically infected (Spearman–Kärber method).

### 2.6. Isolation and Propagation of IBDV B2977 Isolate from a Field Sample

A dead broiler chicken (Chunky Ross308) exhibiting colibacillosis from a chicken farm in the Kyusyu region in 2020 was screened with a virological test (control number B2977). The bursal homogenate that was positive for the IBDV RT-PCR was inoculated into five 10-day-old embryonating SPF hen eggs through the chorioallantoic cavity route. The IBDV was propagated once again by inoculating the homogenate of the embryo into five eggs. The IBDV in the pooled embryo homogenate was designated as the B2977CE2 strain. It was then inoculated orally into twelve 36-day-old SPF chickens. Four chickens were sacrificed at 3, 5, and 7 days post-inoculation (pi) to confirm whether IBDV did not lose its pathogenicity during the egg passages. Bursal homogenates prepared from each sacrificed chicken at 3 and 5 days pi were pooled. IBDV passage in chickens was repeated twice. The third time, bursal homogenate prepared from 4 days pi samples was designated as a master IBDV B2977CE2C3 strain (10^5.2^ EID_50_/μL).

### 2.7. Sequence Alignment and Phylogenetic Tree Analysis of IBDV

The segment A ORF sequence of the IBDVB2977CE2C3 strain was compared with sequences of 70 relevant IBDV strains collected from GenBank. Sequence alignment and phylogenetic tree analysis were performed using the muscle and the maximum likelihood methods with the MEGA11 free software (www.megasoftware.net). The number of nucleotide substitutions was inferred using the Tamura–Nei model by applying Neighbor-Join and BioNJ algorithms with 1000 bootstrap replications. The tree was visualized using FigTree software (version 1.4.4).

### 2.8. Pathogenesis of Novel Variant IBDV B2977CE2C3 Strain in SPF Chickens

Forty-eight 21-day-old SPF chickens were divided into two groups (n = 24 chickens/group) and housed in separate isolators provided with feed and water. Chickens in Group 1 were infected by oral challenge with a dose of 10^4.5^ EID_50_/chicken of IBDV B2977CE2C3 strain, while those in Group 2 were mock-infected as a negative control. At 3, 7, 14, and 21 days pi, six chickens from each group were euthanized and weighed. Blood and five major lymphoid tissues, BF, spleen, thymus, cecal tonsil, and bone marrow, were harvested from the six chickens from each group and assessed for macroscopic lesions and viral loads. The BF samples were weighed to calculate the BF weight to body weight (BF/BW) ratio (%). The scoring for the BF atrophy was determined macroscopically by comparing the major axis of the BF with those isolated from mock-infected chickens: 0  =  more than 90% in size (no noticeable change); +1 = 90> to ≧75% in size; +2 = 75> to ≧65% in size; +3 = less than 65% in size. Viral loads in the five major lymphoid tissues were examined by RT-qPCR. The titrated stock of challenge virus drowned by a known quantity of IBDV cDNA fragment was used to confirm the standard curve. Results were presented as genome copy/μL. Blood was collected from all chickens, and their sera were tested for anti-IBDV antibody titer. The statistical significance of the differences among the different groups was determined using the Student’s *t*-test. Differences were considered significant at *p* < 0.05 (*), *p* < 0.01 (**), and *p* < 0.0001 (***).

### 2.9. Serological Test for Anti-IBDV Antibodies

Sera were prepared from the chicken blood using a standard procedure and were stored at 4 °C until use. Anti-IBDV antibody titers were determined using a commercially available IBDV Antibody Test kit (IDEXX Laboratories, Westbrook, ME, USA) according to the manufacturer’s recommendations. Briefly, heat-inactivated sera were diluted five hundred-fold (1/500) with the sample diluent included in the kit and were used for the ELISA. Then, ELISA optical density values at 650 nm were calibrated with those obtained from positive and negative controls included in the ELISA kit to yield a quantitative result for anti-IBDV antibodies. Sample-to-positive ratio (S/P) values calculated according to the manufacturer’s instructions were used to determine if samples were IBDV-seropositive (S/P > 0.2) or seronegative (S/P ≦ 0.2).

## 3. Results

### 3.1. Detection of Novel Antigenic Variant IBDV Strains in Japan

To clarify the recent status of IBD and the characteristics of prevailing IBDV strains in Japan, broiler chickens sent from the broiler houses between 2014 and 2023 were used to see if IBDV was positive or not by amplifying the DNA fragment containing the HVR using RT-PCR. Totally 122 RT-PCR positive samples were obtained (Table 1). IBDV strains belonging to genogroup A1 classical strains of IBDV were detected in two samples from 2014, four samples from 2015, and one sample from 2016. Very virulent strains of genogroup A3 IBDV were detected in 2014 but were then not detected again during our study. The A4 genogroup contains the distinct IBDV strains that were found sporadically. Members of strains belonging to the genogroup A2 were not found during these years. Interestingly, the sub-genogroup A2d novel antigenic variant strain of IBDV was initially detected in three out of twelve samples (25.0%) in 2017. From 2017 to 2022, 12.5% to 50.0% of the samples were classified into novel antigenic variant strains. These strains remained in the broiler houses in the Kyusyu region. We detected the novel antigenic strains of IBDV in 16 out of 27 samples (59.3%) in 2023. It is noteworthy that one of these was detected in a broiler house on the eastern main island of Japan, outside of the Kyusyu region, suggesting that the novel variant IBDVs have spread in Japan.

### 3.2. Isolation of Novel Variant IBDV

To understand the pathogenesis of the novel antigenic variant IBDV prevailing in Japan, we tried to isolate it from the field samples. Three bursal samples—identified positive for the novel antigenic variant IBDV using RT-PCR and subsequent sequencing followed by a phylogenic tree analysis—were selected as representative samples. Each bursal homogenate was inoculated into three SPF embryonating eggs and incubated for four days. Eggs inoculated with two of the three bursal samples failed to amplify IBDV, but one sample named B2977 obtained in 2020 was successfully amplified in the eggs. Amplified IBDV in the embryonating hen eggs was designated as B2977CE2. The IBDV B2977CE2 strain was then inoculated orally into the 36-day-old chickens. The chicken passage was repeated three times under the same condition, and the third bursal homogenate prepared at 4 days pi was pooled and used as the master viral stock named B2997CE2C3. Atrophy of the BF and multiplication of IBDV were observed in all three passages. A titer of IBDV B2977CE2C3 was calculated to be 10^5.2^ EID_50_/μL.

### 3.3. Segment A Polyprotein ORF Sequence and Phylogenic Tree Analysis

**Figure 1:**
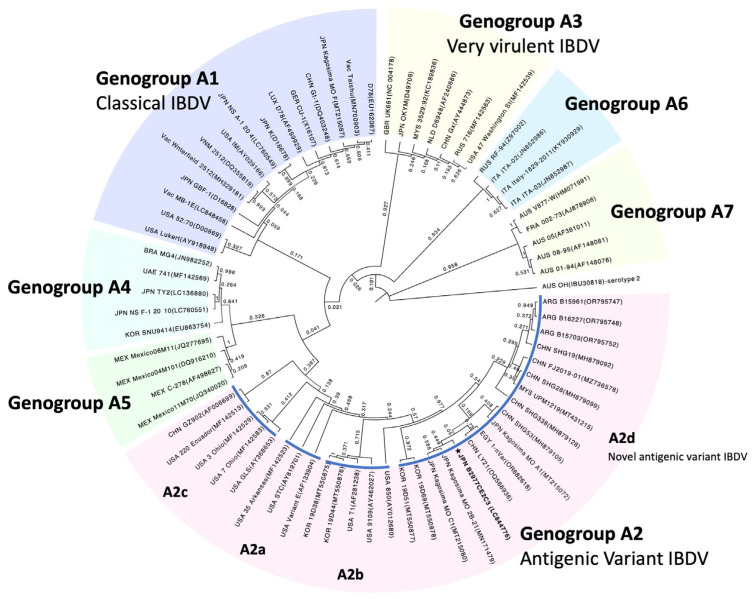
Phylogenic tree of IBDV B2977CE2C3. A phylogenic tree based on the segment A sequence of the IBDV is shown. The IBDV B2977CE2C3 strain is shown in bold with a star. Relevant sequences of IBDV strains collected from GenBank are shown with the DNA database accession number. Seven genogroups, A1 to A7, of the IBDV strains classified using the Maximum Likelihood method are shown. Sub-genogroups (A2a, A2b, A2c, and A2d) further classified from genogroup A2 are also shown.

Segment A VP2-VP4-VP3 polyprotein ORF sequence of the IBDV B2977CE2C3 strain was determined using a primer working method and was registered in the DNA database [GenBank accession number: LC844776]. Segment A polyprotein ORF of the B2977CE2C3 strain was composed of 3039 nucleotides and was classified by phylogenic tree analysis into the sub-genogroup A2d composed of novel antigenic variants (Figure 1). The B2977CE2C3 strain was located in a local branch comprising the Japanese strain, Kagoshima MO 2b-21 [MN171479], the Chinese strains, LY21 [QQ566936] and SHG53.

**Figure 2:**
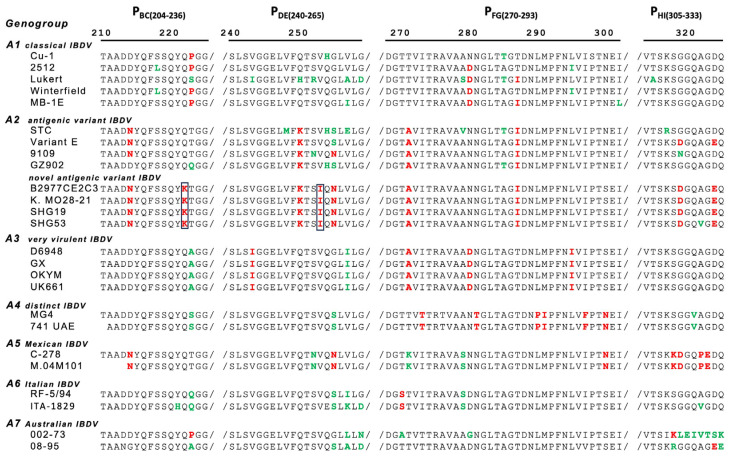
Amino acid substitutions at putative antigenic sites on VP2 projection domains of IBDV. Four P domains, P_BC_, P_DE_, P_FG_, and P_HI_, possessing the putative antigenic sites of VP2, are indicated on the top with the amino acid number. Deduced amino acid sequences in the projection domains of IBDV B2977CE2C3 are compared with those of other strains in different genogroups (A1, A2, A3, A4, A5, A6, and A7). Relevant sequences of IBDV strains were collected from GenBank. A common amino acid found in a genogroup is shown in red. An exceptional amino acid that is not found in other strains within a genogroup is shown in green. Two amino acids (221K and 252I) commonly found in the sub-genogroup A2d strain are shown in the box.

[MH879105], and an Egyptian strain, 1-nVar [OR682618]. Several amino acid substitutions found in the HVR ranging from 211 to 350 aa of the VP2 protein are related to viral pathogenicity and growth ability of the IBDV. The B2977CE2C3 strain has some characteristic amino acids that are common for genogroup A2 variant strains, such as 213N/D and 221K in P_BC_, 270A, 279N, and 286I in P_FG_, and 323D/E in P_HI_ (Figure 2). In particular, amino acid substitutions of 241K in P_BC_ and 252I in P_DE_ are the unique characteristics belonging to the sub-genogroup A2d belonging to novel antigenic variant IBDVs, including the B2977CE2C3 strain.

### 3.4. Experimental Chicken Infection of IBDV B2977CE2C3 Strain

**Figure 3:**
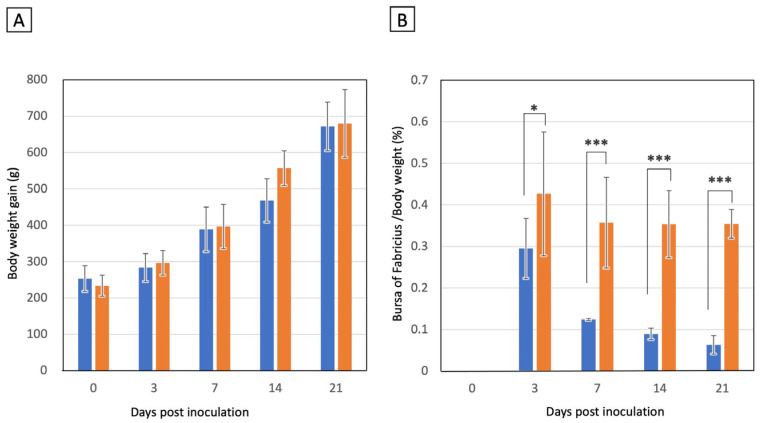
Effects of the infection of novel antigenic IBDV B2977CE2C3 strain on BW and BF. The increase in body weight (**A**) and the body weight/BF weight ratio (**B**) were examined in IBDV B2977CE2C3 infected (blue) and mock-infected chicken (orange) groups. All chickens in both groups were weighed before inoculation (day 0). Six chickens of each group were weighed at 3, 7, 14, and 21 days post-inoculation (pi) and were then sacrificed. The BF was isolated from the sacrificed chickens, and its mass was weighed. The mean value at each measurement point is shown with a bar graph. Symbols * and *** indicate *p* < 0.05 and *p* < 0.001, respectively, calculated using an unpaired *t*-test between the B2977CE2C3 strain-infected and mock-infected groups. Deviation (+/− SD) is shown with error bars on the bar graph.

To assess the pathogenicity of the novel antigenic variant B2977CE2C3 strain in the chickens, 24 SPF chickens were orally inoculated with 10^4.5^ EID_50_ of IBDV (Group 1), and another 24 SPF chickens were mock-inoculated (Group 2) as a negative control. Chickens were weighed at 0, 3, 7, 14 and 21 days pi. The body weight (BW) of chickens increased during the experimental period, and no significant difference in BW was observed between Groups 1 and 2 at any observation points (Figure 3A). Moreover, no apparent symptoms were observed in any chickens during the observation period. The BF was isolated from six chickens at 3, 7, 14, and 21 days pi from each group (see Supplementary Figure S1), and the major axis and the weight of the BF were measured. Macroscopic observations of BF atrophy based on the major axis of the BF are summarized in Table 2. Atrophy of the BF was observed at 3 days pi in two of six chickens (score +1) in Group 1. All six chickens in Group 1 showed atrophy of the BF at 7 days pi (score +1; four chickens, score +2; two chickens, score +3; zero chickens), 14 days pi (score +1; zero chickens, score +2; three chickens, score +3; three chickens), and 21 days pi (score +1; zero chickens, score +2; zero chickens, +3; six chickens). The degree of atrophy was found to increase during the observation period. Chicken BW increased in both Groups 1 and 2 throughout the experimental period regardless of whether IBDV inoculation status, whereas atrophy of the BF progressed gradually in IBDV-inoculated Group 1 chickens. The BF/BW ratios in Group 1 markedly decreased as follows: 0.298 ± 0.072 at 3 days pi, 0.124 ± 0.002 at 7 days pi, 0.089 ± 0.014 at 14 days pi, and 0.063 ± 0.22 at 14 days pi (Figure 3B). In contrast, those of Group 2 were almost constant, considering the deviation, as follows: 0.427 ± 0.149 at 3 days pi, 0.357 ± 0.109 at 7 days pi, 0.363 ± 0.081 at 14 days pi, and 0.354± 0.035 at 14 days pi (Figure 3B). These results demonstrated that the IBDV B2977CE3C2 strain caused severe atrophy of the BF.

### 3.5. IBDV Replication in the Chickens

**Figure 4:**
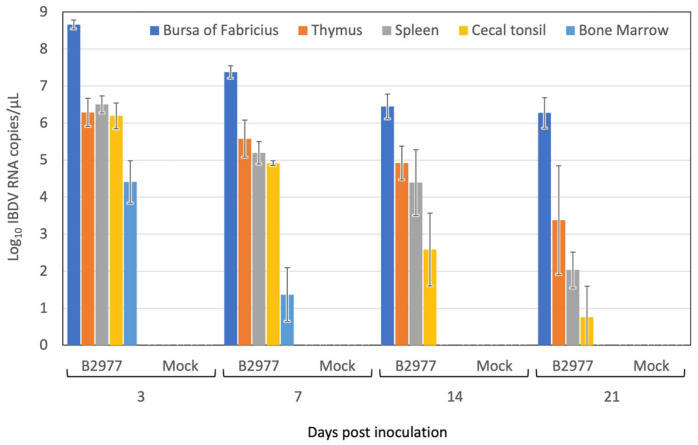
Viral genome copy number in the lymphoid tissues. BF (blue), thymus (orange), spleen (gray), cecal tonsil (yellow), and bone marrow (light blue) were taken from both infected and mock-infected chicken groups at 3, 7, 14, and 21 days post-inoculation (pi). The viral genome copy number in 10% tissue homogenate was measured via quantitative RT-PCR using the quantity known standard as a reference. The vertical axis indicates the natural logarithm of copy number (Log_10_ [copy number]). The horizontal axis indicates days after the B2977CE2C3 strain infection or the mock infection. The mean genome copy number at each measurement point is shown as a bar graph. Deviation (+/− SD) is shown on the mean bar graph.

Besides those in the BF, significant macroscopic lesions were not observed in the major lymphoid tissues, namely the spleen, thymus, cecal tonsil, and bone marrow, which were harvested from the six chickens from both Groups 1 and 2 during all experimental periods. Viral RNA was extracted from the 10% homogenate of individual lymphoid tissue samples and measured using the quantitative RT-PCR to assess the viral loads. The viral RNA copy number showed a clear contrast with the highest copy number of IBDV RNA (10^8.66±0.12^ copies/μL) being detected in the BF at 3 days pi, but it decreased to 10^7.34±0.17^ copies/μL at 7 days pi, 10^6.45±0.34^ copies/μL at 14 days pi, and 10^6.27±0.41^ copies/μL at 21 days pi (Figure 4). This indicated that IBDV B2973E2C3 replicated in the BF during the early stage of infection (less than 3 days pi). The IBDV genome was continuously present, even at 21 days pi, but the copy number of the genome decreased by almost one-hundredth, probably due to the dying off of host lymphoid cells in the BF. A similar copy number of the IBDV genome (10^6^ copies/μL) was found in the thymus, spleen, and cecal tonsil at 3 days pi. The copy number of the IBDV in the thymus decreased gradually with time and reached one-hundredth at 21 days pi. The decrease in copy number of the IBDV genome in the spleen and cecal tonsil was much greater than that in the thymus. The copy number was reduced to one-thousandth in the spleen and below in the cecal tonsil at 21 days pi. In the bone marrow, 10^4.4±0.58^ copies/μL of the IBDV RNA genome was detected at 3 days pi, while the IBDV genome decreased to less than 100 copies/μL from 14 days onwards.

### 3.6. Anti-IBDV Antibodies in the Chickens

**Figure 5:**
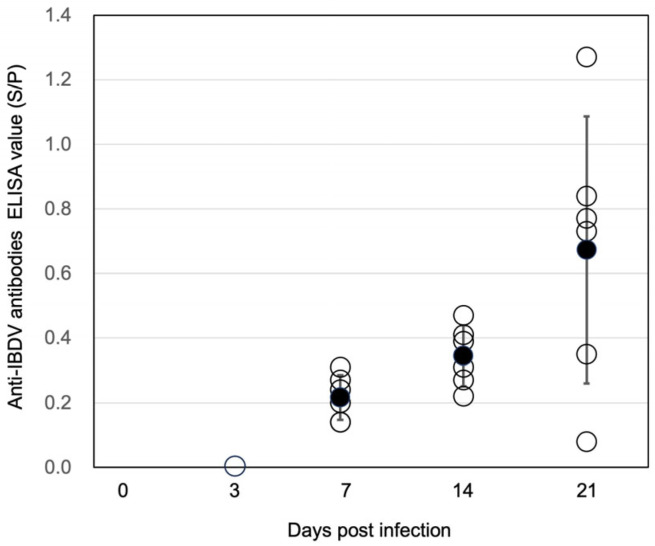
Anti-IBDV serum titers of IBDV B2977CE2C3 infected chickens. The vertical axis indicates anti-IBDV antibody ELISA titers (S/N). The horizontal axis indicates days post-infection. Six sera are shown individually by open circles. The closed circle indicates the mean anti-IBDV antibody ELISA titer. Deviation (+/− SD) is shown on the closed circle representing the mean value. The cutoff value is 0.2. Since none of the samples at 3 days post-infection produced an anti-IBDV ELISA value (S/P), an open circle is representatively shown at 0.0 on the vertical axis.

As the IBDV B2977CE3C2 strain caused significant damage in the central B lymphocyte organ, BF, we attempted to identify whether the B cell immune response against the inoculated IBDV was raised or not. Chicken sera were prepared from blood taken during the IBDV experimental infection experiment and were used for the measurement of anti-IBDV serum titer using the commercially available ELISA kit. No IBDV-positive serum was detected at 3 days pi, indicating an absence of maternal antibodies in the chickens. However, four of six sera gave slightly positive (S/P > 0.2) results at 7 days pi (Figure 5), and six of six sera gave slightly positive results at 14 days pi. Although one out of six sera was negative against IBDV, four sera showed highly positive titers, and one showed a weak positive titer. These results indicated that the BF function producing the specific immunoglobulin against the antigen was at least partly maintained, even in a BF with severe atrophy.

## 4. Discussion

The novel antigenic variant IBDV was detected in central China in the mid-2010s [26] and has become widespread in other parts of China since 2017 [24]. In our collection, a novel antigenic variant of IBDV was first detected in a case isolated in the western part of Japan in 2017. This concurs with a primary case report of the novel antigenic variant IBDV occurring in Japan by Myint et al. [28]. Considering a South Korean report indicating that five novel antigenic variant IBDVs were identified through passive surveillance in 2019 [27], the Japanese novel antigenic variants were thought to derive from China rather than South Korea. This hypothesis was supported by a phylogenic tree analysis, which showed a closer relationship between Japanese isolates and Chinese isolates than between Japanese isolates and Korean isolates (Figure 1).

Recently, the number of novel antigenic variant IBDV cases has increased and expanded in Japan (Table 1). Though the initial case reports were restricted to the western part of Japan, novel antigenic variant IBDVs have since been found in the eastern part of Japan. This indicates the spread of the novel antigenic variant IBDVs in Japan, even though IBD has been controlled with IBD vaccines. In this study, we isolated the IBDV B2977CE3C2 strain from a field sample prepared from the IBD case that occurred in the western part of Japan in 2020. The polyprotein gene sequence of segment A showed a close relationship with the novel antigenic variant IBDV strain isolated in Japan, China, Egypt, and Argentina, indicating the possibility of the novel variant IBDV spreading over far eastern Asia. Primary vaccine failure for IBD was reported in China [36]. Several amino acid changes specific to the novel antigenic variants are found in the projection region of the VP2 protein, and they are thought to be responsible for the immune escape of the novel antigenic variant IBDVs [37,38]. It was indeed shown that some monoclonal antibodies that can react with vvIBDVs do not react with the Chinese novel antigenic variant strain SHG19 [25]. Furthermore, the neutralization index studied using DT40 cells was demonstrated to be 0.64 or less between the SH1G19 strain and vvIBDV strains [38]. It was suggested that booster immunization of the usual IBD vaccines could be used to overcome the amino acid differences in the projection domain of the VP2 protein [39].

Experimental IBDV B2977CE3C2 infection in the SPF chickens showed the unique feature of novel antigenic variant IBDV: neither lethality nor apparent macroscopic lesions, except in the BF, were present. Moreover, BW gain in infected chickens was almost similar to that of chickens in the negative control group. In contrast, experimental infection with the vvIBDV caused death in seven of fifteen chickens (46.7%) for the 89163 strain [40] and in four of five chickens (80.0%) for the HLJ0504 strain [41]. However, severe atrophy of the BF (shown by the BF/BW ratio) caused by IBDV B2977CE3C2 (less than 0.1 at 14 and 21 days pi) was similar to that caused by very virulent strains [41] but did not occur in classical IBDV infection [42]. The highest copy number of the IBDV genome was observed in the BF at 3 days pi., and the copy number of the IBDV genome declined one-tenth at 7 days pi and one-hundredth at 14 and 21 days pi, probably due to the consumption of permissive cells. Although no macroscopic lesions were observed in the thymus, spleen, or cecal tonsil, the IBDV genome was detected in these organs as well. Since the amount of the present genome could not simply be explained by leaking IBDV-infected lymphocytes from the BF, it is probable that some of the cells in these organs were infected with IBDV. This remains to be investigated in subsequent microscopic and pathological research.

Humoral immune response against IBDV was observed even though the BF was severely damaged. The anti-IBDV antibodies measured at 7 days pi were considered the production of IgM, and those measured later were considered the production of IgG. This indicated that the occurrence of class-switch events in the immunoglobulin gene is common, even in an IBDV-infected BF. The poultry houses with detected IBDV B2977 sequence reported that some chickens over 30 days old exhibited a loss of motor function, a reduction in weight gain, and death from colibacillosis. This was possibly caused by a secondary infection that occurred as a result of the immune incompetence caused by the novel antigenic variant IBDV infection. While this study ran for 21 days after the IBDV infection and the humoral reaction of chickens against IBDV was observed, a case of IBD in the field suggests that the humoral immune dysfunction of the BF occurred to a greater extent and lasted longer in the field chickens compared to the experimentally infected chickens. Experimental precautions should account for the fact that while our experiment was conducted using SPF egg-laying chickens, the novel antigenic variant IBDV cases in the fields largely occur in broiler chickens. It is possible that the genetic makeup of chickens, such as egg-laying chickens and broiler chickens, influences their reaction to IBD. However, it is worth noting that SPF white leghorn egg-laying chickens were more susceptible to vvIBDV than SPF white broiler chickens, showing more severe pathologic changes in lymphoid tissues and kidneys, as well as clinical signs and mortality [43].

Experimental infection of novel antigenic variant IBDV SHG19 strain in commercial broilers was demonstrated to induce hemagglutination inhibition (HI) antisera against Newcastle disease (ND) virus following the immunization with the ND vaccine at 4 days post SHG19 inoculation, although statistical significance of HI titer was observed [44]. Collectively, these results indicate that care should be taken when applying experimental results to field situations. In future research, we aim to confirm the pathogenesis of the novel antigenic variant IBDV and investigate the protection efficacy against novel antigenic variant IBDV from the maternal or chick antibodies induced by the commercially available vaccines with the antigenic mismatch.

Viral antigenicity of IBDV is dominated by the segment A RNA genome coding the VP2 protein. In contrast, the viral capacity of replication in the host cells is primarily dominated by the Segment B RNA genome coding the VP1 replicase, implying that the VP1 protein is involved in viral pathogenesis. In the classification of IBDV, the genotype based on the combination of segments A and B, like A1B1, has been suggested [32]. In the case of novel antigenic variant IBDV, A2dB1b was reported [30]. In the following study, we will identify the B segment-based genogroup of our novel antigenic variant strain.

## Figures and Tables

**Table 1:** Detection of novel antigenic variant IBDV strains in Japan.

Fiscal Year	Total Test Numbers *	Genogroup A1	Genogroup A2		Genogroup A3	Genogroup A4	Novel AntigenicVariants/Total (%)
		Classical	Antigenic Variant	Novel Antigenic Variant	Very Virulent	Distinct	
2014	5	2	0	0	2	1	0.0
2015	5	4	0	0	0	1	0.0
2016	1	1	0	0	0	0	0.0
2017	12	9	0	3	0	0	25.0
2018	16	10	0	6	0	0	37.5
2019	6	3	0	3	0	0	50.0
2020	8	6	0	1	0	1	12.5
2021	23	12	0	6	0	5	26.1
2022	19	15	0	3	0	1	15.8
2023	27	9	0	16	0	2	59.3
	122	71	0	38	2	11	

* RT-PCR-positive field samples from broiler chickens with suspected IBD in farms were used for analysis. In addition to the prevalent field IBDV strains, live attenuated IBD vaccines used on chicken farms may also be included in the survey. Genogroup typing of IBDV was performed using a phylogenic tree analysis following RT-PCR amplification and sequencing of the HVR of the *VP2* gene. No vaccines belonging to the genogroup A2 are used in Japan.

**Table 2:** Atrophy of the BF in chickens challenged with novel antigenic variant IBD.

	IBDV Infected Chickens Group 1				Mock Infected Chickens Group 2			
BF Atrophy Score *	3	7	14	21 Days pi	3	7	14	21 Days pi
0	4	0	0	0	6	6	6	6
+1	2	4	0	0	0	0	0	0
+2	0	2	3	0	0	0	0	0
+3	0	0	3	6	0	0	0	0
Mean score ^#^	0.33	1.33	2.50	3.00	0.00	0.00	0.00	0.00

* Atrophy of the BF in six chickens challenged with novel antigenic variant IBDV or mock challenged (the control) was observed at 3, 7, 14, and 21 days post-infection. The number indicates the number of chickens that showed each BF atrophy score. # The mean score is calculated by multiplying the BF atrophy score by the number of chickens and dividing by six; this represents the total number of chickens sacrificed on each observation day.

## Data Availability

The datasets analyzed during the current study are available from the corresponding author upon reasonable request.

## Supplementary Materials

The following supporting information can be downloaded at: https://www.mdpi.com/article/10.3390/pathogens13121141/s1, Figure S1: BF atrophy caused by the infection of novel antigenic IBDV B2977CE2C3 strain.
