# Supplementary figures and images for: Novel Antigenic Variant Infectious Bursal Disease Virus Outbreaks in Japan from 2014 to 2023 and Characterization of an Isolate from Chicken
Source: Pathogens. 2024 Dec 23;13(12):1141. doi: 10.3390/pathogens13121141 (PMC11678736; doi:10.3390/pathogens13121141)

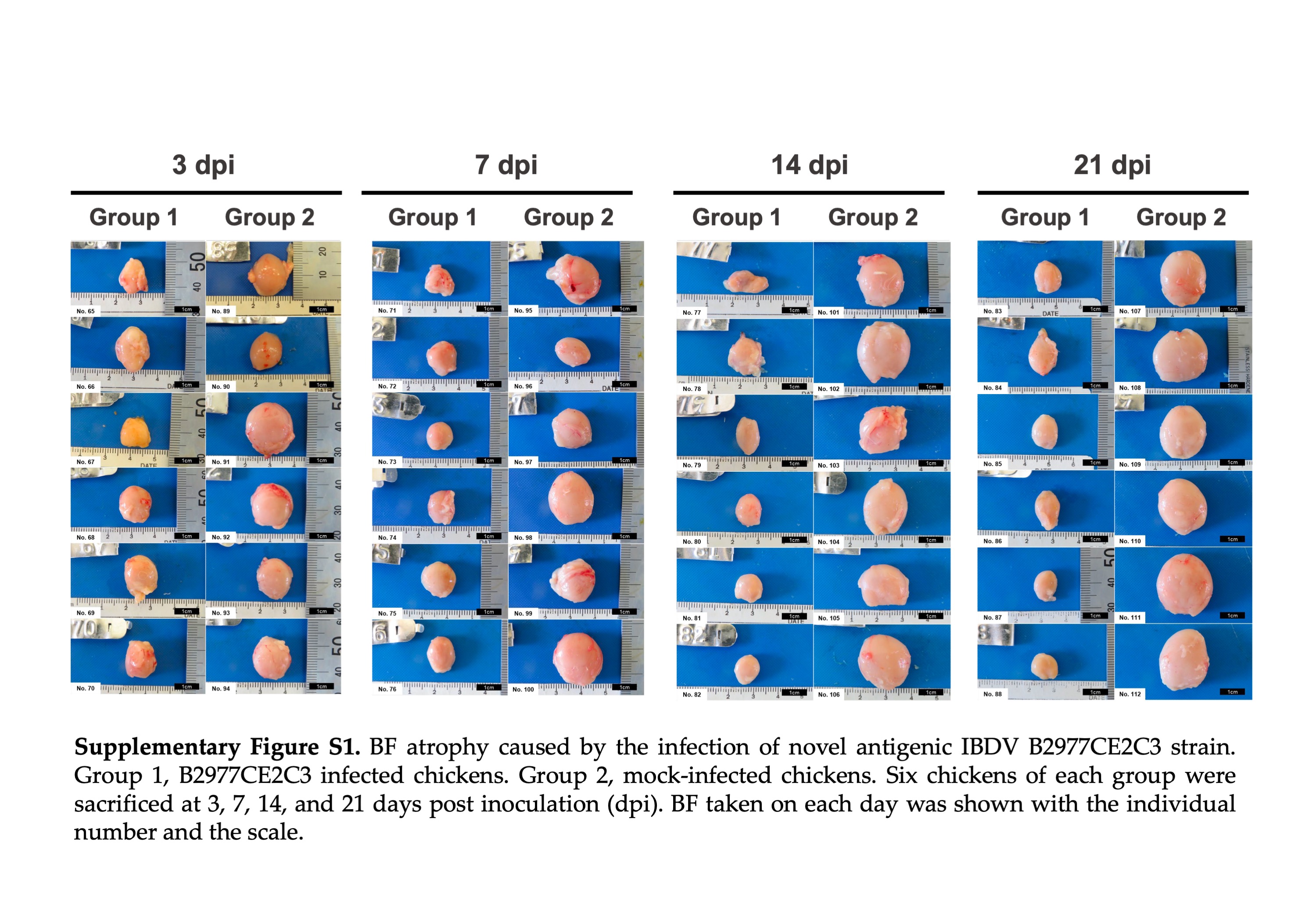

Supplement: Supplementary file 1 [file pathogens-13-01141-s001.zip › pathogens-3358063-supplementary.jpg]
